# Retinal microvasculature features in patients with Behcet’s disease: a systematic review and meta-analysis

**DOI:** 10.1038/s41598-021-04730-6

**Published:** 2022-01-14

**Authors:** Kai-bao Ji, Zhe Hu, Qing-lin Zhang, Hai-feng Mei, Yi-qiao Xing

**Affiliations:** 1 Deparment of Ophthalmology, Renmin Hospital of Wuhan University, Wuhan, China; 2 Department of Ophthalmology, Huangshi Central Hospital, Edong Healthcare Group, Affiliated Hospital of Hubei Polytechnic University, Huangshi, China

**Keywords:** Biomarkers, Diseases, Medical research

## Abstract

This meta-analysis aimed to analyze retinal microvasculature features in eyes with Behçet’s disease (BD) using optical coherence tomography angiography (OCTA). Electronic databases, including PubMed, Web of Science, Embase, and Cochrane Library, were comprehensively searched for published studies comparing retinal microvasculature characteristics between eyes with BD and controls. Continuous variables were calculated using the mean difference (MD) with 95% confidence interval (CI). Review Manager software (version 5.30) was used to conduct statistical analysis. A total of 13 eligible studies involving 599 eyes with BD and 622 control eyes were included in the meta-analysis. The pooled results showed that the macular whole enface superficial and deep vessel density (VD) values measured by OCTA were significantly lower in eyes with BD than in control eyes (superficial VD: MD = − 3.05, *P* < 0.00001; deep VD: MD = − 4.05, *P* = 0.0004). The foveal superficial and deep VD values were also significantly lower in the BD group than in the control group (superficial VD: MD = − 1.50, *P* = 0.009; deep VD: MD = − 4.25, −  = 0.03). Similarly, the analysis revealed a significant reduction in the parafoveal superficial and deep VD in eyes with BD than in control eyes (superficial VD: MD =  − 3.68, *P* < 0.00001; deep VD: MD = − 4.95, *P* = 0.0007). In addition, the superficial and deep foveal avascular zones (FAZs) were significantly larger in patients with BD than in controls (superficial FAZ: MD = 0.06, *P* = 0.02; deep FAZ: MD = 0.12, *P* = 0.03). The present meta-analysis found that macular whole enface VD, foveal VD, and parafoveal VD were lower in eyes with BD, and the FAZ was larger in patients with BD. The findings suggest that OCTA can assist clinicians in diagnosing and monitoring the status of patients with BD.

## Introduction

Behçet’s disease (BD) is a chronic multisystem inflammatory disease characterized by relapsing oral and genital ulcers, ocular lesions, skin lesions, and vascular inflammation^1,2^. BD is particularly common in Middle Eastern countries^3^, and the highest prevalence of BD has been estimated to be 420 per 100,000 individuals in Turkey^4^. Although the underlying pathological mechanisms remain unknown, genetic and immunological factors, as well as environmental agents, have been implicated in the onset of BD^5^. Prior studies showed that ocular involvement occurred in 40%–70% of patients with BD^6,7^. Typical ocular involvements include non-granulomatous panuveitis and retinal vasculitis^8^. Retinal vasculitis may lead to macular edema if untreated, resulting in severe loss of vision^9^. Therefore, early detection and timely treatment are critical for visual prognosis^8^.

Currently, fundus fluorescein angiography (FFA) has become the gold standard for evaluating retinal vasculitis or macular edema in patients with BD^10^. However, FFA is an invasive procedure because of the need for intravenous dye injection and cannot quantify retinal microvascular structures at different layers^11,12^. Notably, optical coherence tomography angiography (OCTA) is a rapid, non-invasive, high-resolution, novel imaging technique that can quantitatively evaluate retinal and choroidal vessel structures without the need for fluorescein dye injection^13^, and it has been utilized to investigate retinal microvascular changes in various retinal vascular diseases and uveitis^14,15^. Some studies using OCTA showed that both superficial and deep foveal vessel densities (VDs) were significant lower in eyes with BD^16,17^, and lower foveal VD was positively correlated with visual acuity^18^. However, other studies found no difference in foveal superficial vascular density between eyes with BD and controls^19,20^. Given these inconsistent results, further meta-analyses of published studies should be performed. Indeed, to the best of our knowledge, no meta-analysis has comprehensively evaluated retinal microvasculature features related to eyes with BD.

Therefore, we conducted the present meta-analysis to determine retinal microvasculature features in participants with BD and provide more evidence for early identification and therapeutic intervention in patients with BD.

## Methods

### Search strategy

This meta-analysis was carried out in compliance with the Preferred Reporting Items for Systematic reviews and Meta-Analyses (PRISMA) guidelines^21^, and ethical approval was not required. Electronic databases including PubMed, Embase, Cochrane Library, and Web of Science were comprehensively searched to identify qualified literature from inception to April 8, 2021. The following search terms were used: ((((OCTA) OR (OCT angiography)) OR (optical coherence tomography angiography)) OR (optical coherence tomographic angiography)) AND (((Behcet’s disease) OR (Behcet disease)) OR (Behcet’s syndrome)) OR (BD)). Articles published in English were considered eligible. Any disagreements were resolved via discussion with two authors (K-B. J. and Z. H.) or with the third author (Y-Q. X.). Detailed process of electronic search strategy has been showcased in Supplementary Table S1.

### Inclusion criteria

Eligibility criteria were based on the PICOS (population, intervention, control, outcome and study design) principles. The inclusion criteria were as follows: (1) studies on BD patients who fulfilled the diagnostic criteria initiated by the International Study Group for Behçet’s Disease^22^, BD in the inactive phase, and evidence of posterior segment involvement^23,24^; (2) studies comparing retinal microvasculature features between patients with BD and healthy controls using OCTA; (3) studies in which individuals with no ocular or systemic abnormalities served as healthy controls; (4) studies in which primary outcomes included macular whole enface superficial and deep VD, foveal superficial and deep VD, parafoveal superficial and deep VD, and superficial and deep foveal avascular zone (FAZ); and (5) case–control studies of human participants.

Studies were excluded if (1) they were duplicate studies; (2) they were reviews, case reports, animal studies, conference abstracts, comments, or posters; (3) they reported insufficient data or data could not be extracted; and (4) the study objective did not meet the inclusion criteria.

### Data extraction

Two authors (K-B. J. and Z. H.) independently collected data from the selected studies, and any discrepancies were resolved via discussion. The following data were extracted from the included studies: name of the first author, year of publication, country, type of study, mean age, sample size, OCTA device, outcomes, diagnostic criteria of BD, and quality scores.

### Quality assessment

Quality assessment of the included studies was conducted using the Newcastle–Ottawa Scale, which included subject selection (4 points), subject comparability (2 points), and exposure assessment (3 points), with a score range of 0 to 9 points^25,26^. Studies with a summary score of 5 or higher were considered to be of better quality^26^.

### Statistical analysis

In this meta-analysis, the Review Manager (RevMan) software (version 5.30) (Cochrane Collaboration, Oxford, UK) was performed to analyze the extracted data. Continuous variables were presented as the mean ± standard deviations (SDs), and mean differences (MDs) with its 95% confidence interval (CI) was evaluated for pooled effect. The sample mean and SD were calculated as previously^27^. Heterogeneity among studies was assessed using Chi-square statistic test and I^2^ statistic test. I^2^ values of 25%, 50%, and 75% were regarded as mild, moderate, and high heterogeneity, respectively. A fixed-effect model was used if there was no significant heterogeneity among studies; otherwise, a random-effect model was employed. Funnel plots were utilized to evaluate the publication bias. *P* < 0.05 was considered statistically significant among studies.

## Results

### Search results

**Figure 1:**
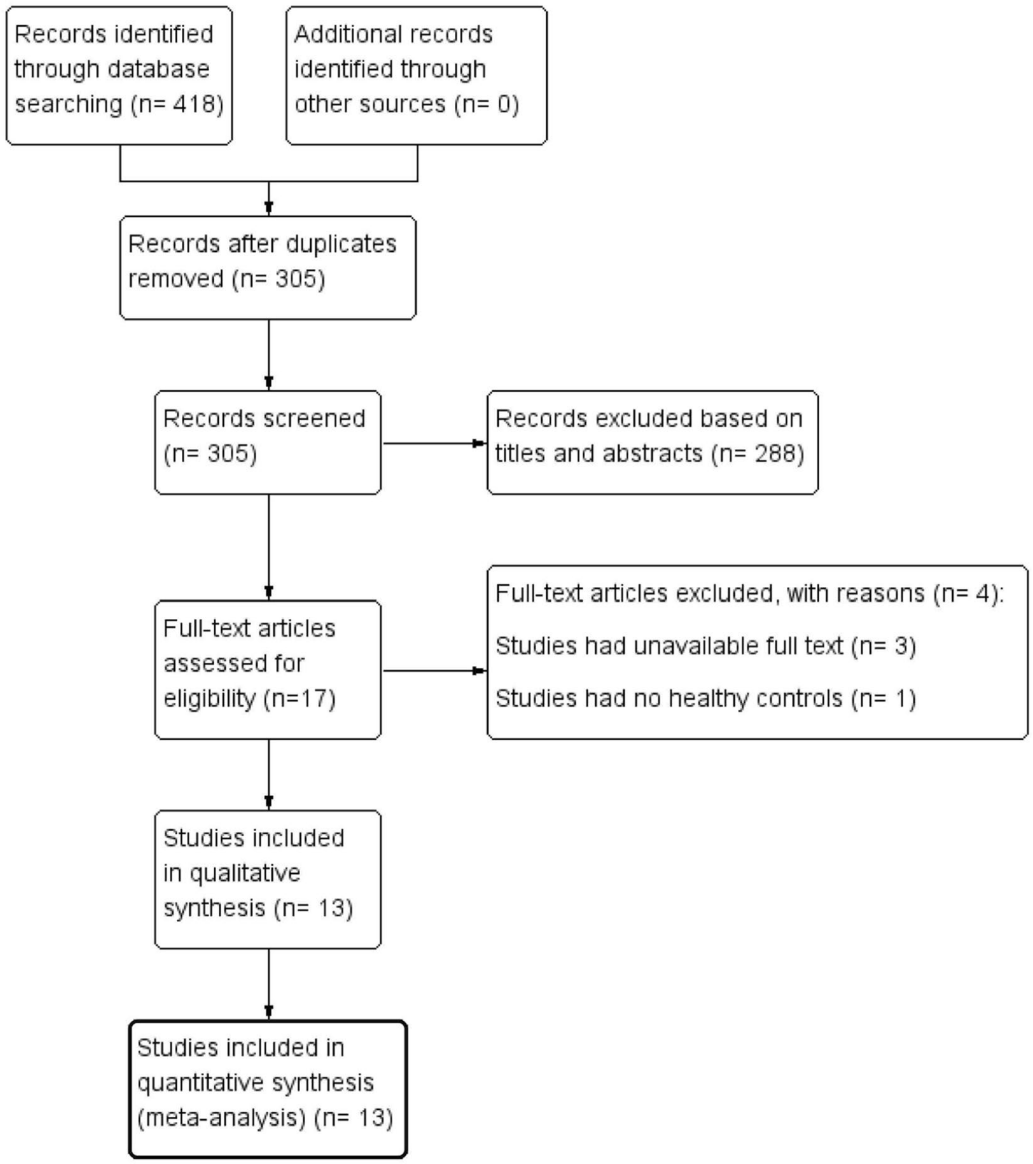
Flow diagram of study selection in the meta-analysis.

The literature retrieval and screening processes are shown in Fig. 1. A total of 418 potentially related articles were initially obtained from all databases (PubMed: 185; Embase: 157; Web of Science: 64; Cochrane Library: 12), of which 113 duplicated publications were excluded. In addition, 288 studies were excluded after the titles and abstracts were carefully screened. Moreover, in the remaining 17 studies, three studies had unavailable full text, and one study had no control group. Finally, 13 articles^16–20,28–35^, involving 599 eyes with BD and 622 control eyes, were included in our meta-analysis.

Table 1 summarizes general characteristics of the 13 included studies, and the results of their quality assessments are displayed in Table 2.

**Table1:** General characteristics of the eligible studies included in the meta-analysis.

Study	Country	Age (years)	Design	Number of eyes	OCTA device	Scan size (mm^2^)	Primary outcomes	Diagnostic criteria
Goker et al.^16^	Turkey	37.5 ± 14.3 39.2 ± 14.9	Cross-sectional study	BD Cases: 22 Controls: 28	Optovue	6 × 6 in macular	Macular whole enface superficial and deep VD, Foveal superficial and deep VD, Parafoveal superficial and deep VD, Superficial FAZ	ISGBD
Çömez et al.^17^	Turkey	39.81 ± 8.93 41.21 ± 9.87	Case–control study	BD Cases: 42 Controls: 40	Optovue	6 × 6 in macular	Macular whole enface superficial and deep VD, Superficial and Deep FAZ	ISGBD
Türkcü et al.^18^	Turkey	33.8 ± 4.51 32.6 ± 4.06	Case–control study	BD Cases: 60 Controls: 62	Optovue	3 × 3 in macular	Macular whole enface superficial and deep VD, Foveal superficial and deep VD, Superficial and Deep FAZ	ISGBD
Karalezli et al.^19^	Turkey	38.50 ± 14.30 40.20 ± 14.10	Case–control study	BD Cases: 56 Controls: 50	Optovue	6 × 6 in macular	Macular whole enface superficial and deep VD, Foveal superficial and deep VD, Parafoveal superficial and deep VD	ISGBD
Emre et al.^20^	Turkey	39.44 ± 13.56 38.1 ± 6.76	Case–control study	BD Cases: 26 Controls: 30	Optovue	6 × 6 in macular	Macular whole enface superficial and deep VD, Parafoveal superficial and deep VD	ISGBD
Accorinti et al.^28^	Italy	38.7 ± 13.2 35.7 ± 7.8	Cross-sectional study	BD Cases: 15 Controls: 15	Zeiss	6 × 6 in macular	Macular whole enface superficial and deep VD	ISGBD
Koca et al.^29^	Turkey	40.54 ± 9.4 41.59 ± 8.9	Case–control study	BD Cases: 94 Controls: 53	Optovue	3 × 3 in macular	Macular whole enface superficial and deep VD, Parafoveal superficial and deep VD	ISGBD
Aksoy et al.^30^	Turkey	38 ± 7.1 37 ± 8.2	Case–control study	BD Cases: 35 Controls: 30	Optovue	6 × 6 in macular	Foveal superficial and deep VD, Parafoveal superficial and deep VD, Superficial and Deep FAZ	ISGBD
Eser-Ozturk et al.^31^	Turkey	35.7 ± 11.65 40.1 ± 9.08	Cross-sectional study	BD Cases: 42 Controls: 38	Topcon	3 × 3 in macular	Parafoveal superficial and deep VD	ISGBD
Smid et al.^32^	Netherland	49.5 ± 12.12 44 ± 13	Cross-sectional study	BD Cases: 46 Controls: 22	Heidelberg	6 × 6 in macular	Parafoveal superficial and deep VD	ISGBD
Cheng et al.^33^	China	38.7 ± 9.5 37.5 ± 6.5	Case–control study	BD Cases:19 Controls: 25	Optovue	3 × 3 in macular	Macular whole enface superficial and deep VD, Superficial and Deep FAZ	ISGBD
Yilmaz et al.^34^	Turkey	38.05 ± 11.52 39.6 ± 9.6	Cross-sectional study	BD Cases:40 Controls: 30	Optovue	6 × 6 in macular	Foveal superficial and deep VD, Parafoveal superficial and deep VD,	ISGBD
Pei et al.^35^	China	29.0 (26.0, 36.0) 29.0 (27.0, 40.75)	Case–control study	BD Cases:102 Controls: 124	Optovue	3 × 3 in macular	Parafoveal superficial and deep VD	ISGBD

*VD* vessel density, *FAZ* Foveal avascular zone, *ISGBD* International Study Group for Behçet’s disease.

**Table2:** NOS quality assessment for the included studies.

Methodological item for non-randomized studies (No. 1–8)	Goker et al.^16^	Çömez et al.^17^	Türkcü et al.^18^	Karalezli et al.^19^	Emre et al.^20^	Accorinti et al.^28^	Koca et al.^29^	Aksoy et al.^30^	Eser-Ozturk et al.^31^	Smid et al.^32^	Cheng et al.^33^	Yilmaz et al.^34^	Pei et al.^35^
1. Is the Case Definition Adequate?	1	1	1	1	1	1	1	1	1	1	1	1	1
2. Representativeness of the Cases	1	1	1	1	1	1	1	1	1	1	1	1	1
3. Selection of Controls	0	0	0	0	0	0	0	0	0	0	0	0	0
4. Definition of Controls	1	0	1	1	1	1	1	1	0	0	1	0	1
5. Comparability of Cases and Controls on the Basis of the Design or Analysis	2	2	2	2	2	2	2	2	2	2	2	2	2
6. Ascertainment of Exposure	1	0	1	1	1	1	0	0	1	0	0	0	1
7. Same method of ascertainment for cases and controls	1	1	1	1	1	1	1	1	1	1	1	1	1
8. Non-Response Rate	0	0	0	0	0	0	0	0	0	0	0	0	0
Total score	7	5	7	7	7	7	6	6	6	5	6	5	7

NOS: Newcastle–Ottawa scale. The selection area included Nos. 1–4, which was up to one score in one question; The comparability area included No. 5, which was up to 2 scores in the question; The exposure area included Nos. 6–8, which was up to one score in one question. The total score was 9.

### Macular whole enface VD analysis in patients with BD and controls

**Figure 2:**
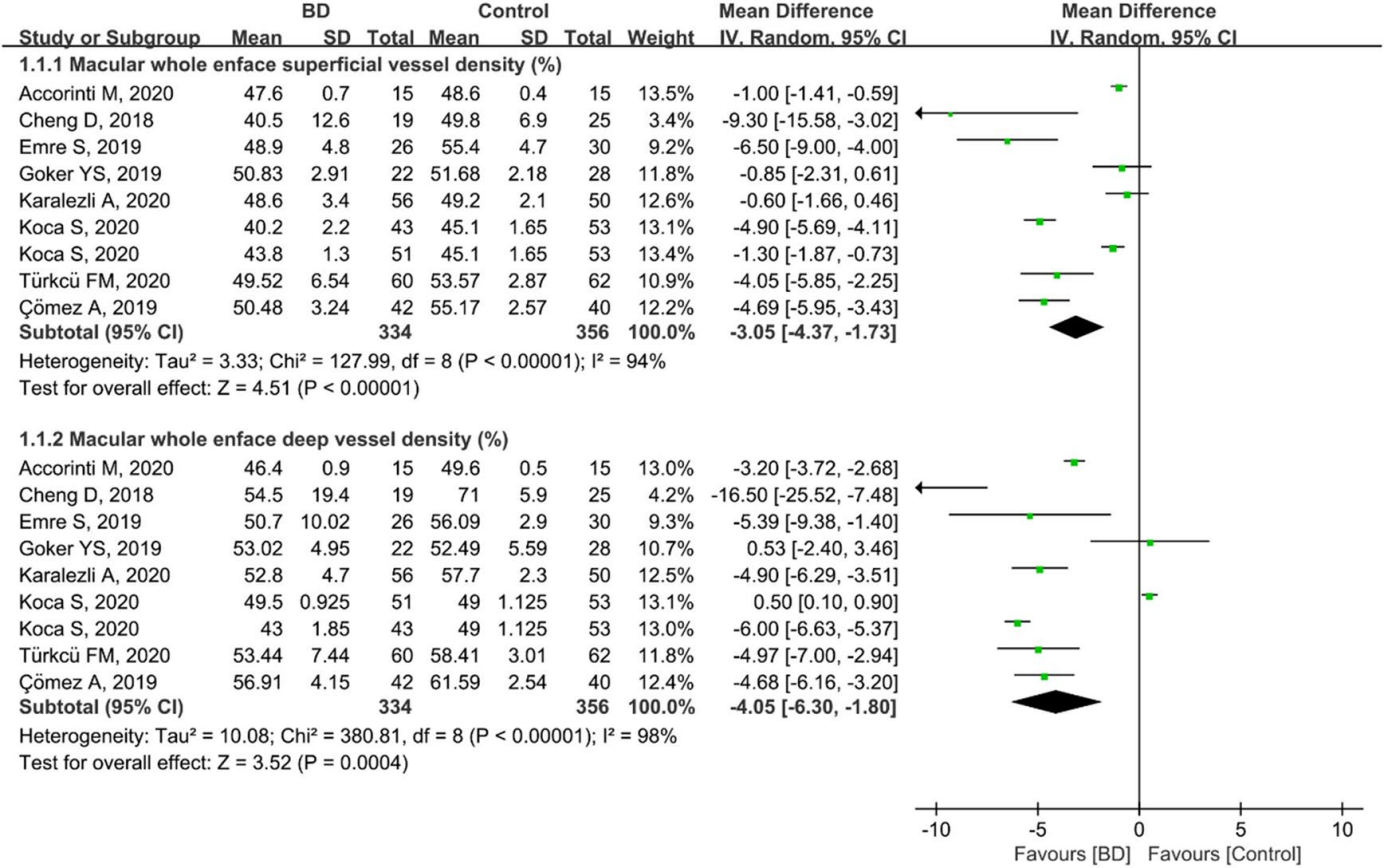
Forest plot for macular whole enface superficial and deep vessel density between BD and control groups.

Eight studies including 690 eyes (334 eyes in the BD group and 356 eyes in the control group) reported the macular whole enface superficial and deep VD. The pooled MD in macular whole enface superficial VD between the BD and control groups was − 3.05 (95%CI: − 4.37 to − 1.73, *P* < 0.00001; Fig. 2), with significant heterogeneity across studies (chi^2^ = 127.99, *P* < 0.00001, I^2^ = 94%; Fig. 2), indicating that the macular whole enface superficial VD was significantly lower in patients with BD than in controls. In addition, the pooled MD in macular whole enface deep VD was − 4.05 (95%CI: − 6.30 to − 1.80, *P* = 0.0004; Fig. 2), revealing that macular whole enface deep VD was also significantly lower in BD patients than in controls. Although a significant difference was found between these two groups, there was high heterogeneity among the studies for this outcome (chi^2^ = 380.81, *P* < 0.00001, I^2^ = 98%; Fig. 2).

**Figure 3:**
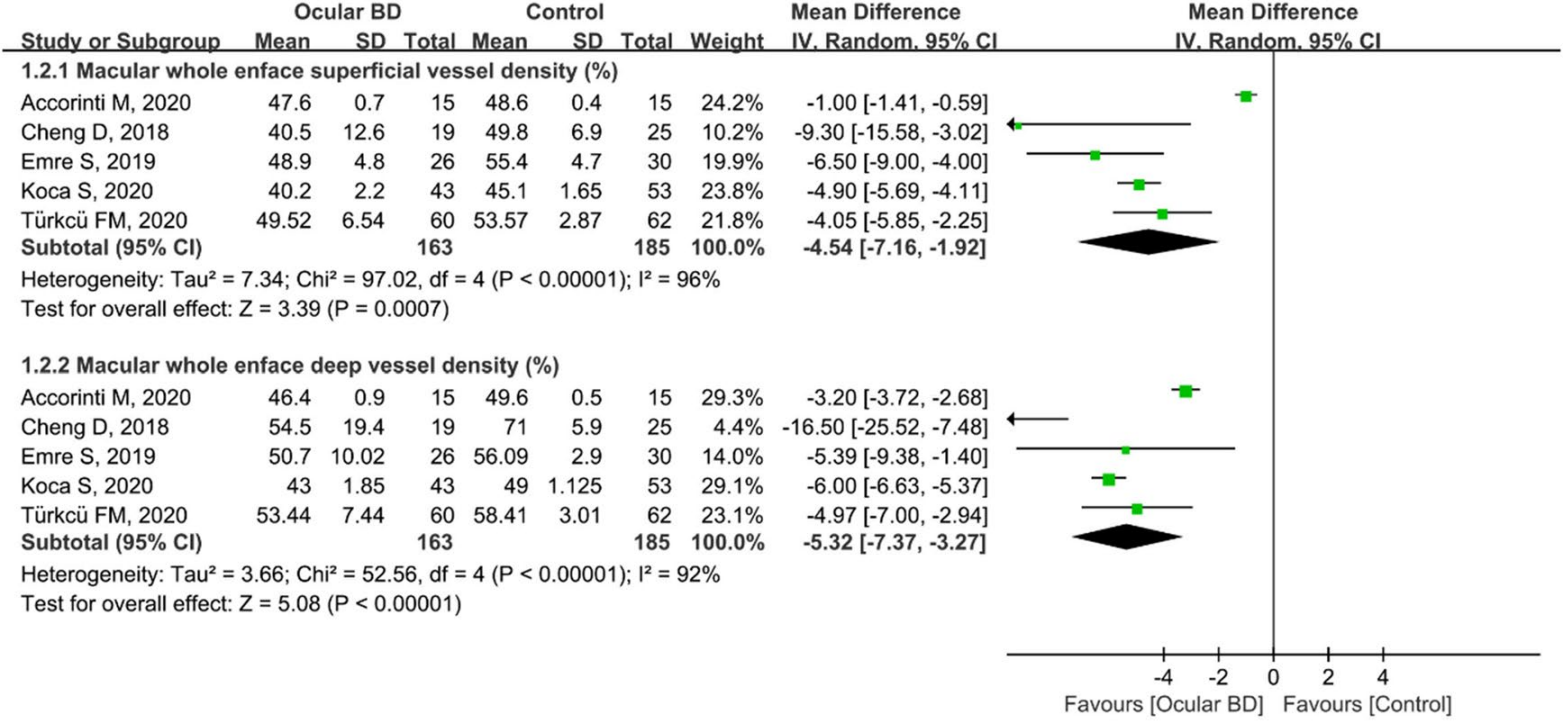
Forest plot analysis of macular whole enface superficial and deep vessel density between eyes with ocular BD and the controls.

Moreover, five studies including 348 eyes (163 in the ocular BD group and 185 in the control group) reported superficial and deep macular VD in their participants. The summary MD in the macular whole enface superficial VD between these two groups was − 4.54 (95%CI: − 7.16 to − 1.92, *P* = 0.0007; Fig. 3), demonstrating that macular whole enface superficial VD was substantially lower in patients with ocular BD; however, high heterogeneity existed across the studies (chi^2^ = 97.02, *P* < 0.00001, I^2^ = 96%; Fig. 3). Subgroup results also showed that macular whole enface deep VD was significantly lower in the ocular BD group than in the control group (MD = − 5.32, 95%CI: − 7.37 to − 3.27, *P* < 0.00001; Fig. 3), with substantial heterogeneity among the studies (chi^2^ = 52.56, *P* < 0.00001, I^2^ = 92%; Fig. 3).

**Figure 4:**
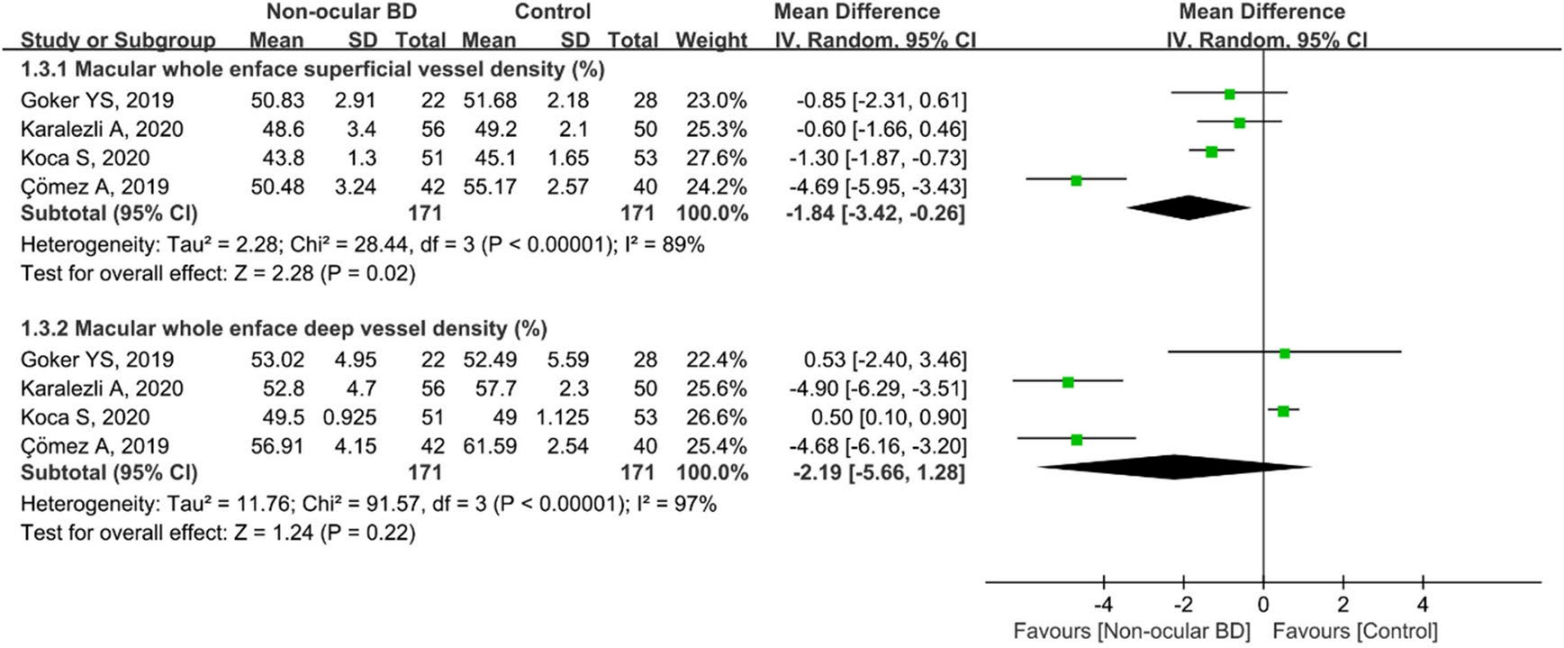
Forest plot analysis of macular whole enface superficial and deep vessel density in non-ocular BD patients and controls.

Furthermore, four studies including 342 eyes (171 in the non-ocular BD group and 171 in the control group) analyzed macular whole enface VD in both the superficial and deep layers. Compared with the control group, the non-ocular BD group showed significantly lower macular whole enface superficial VD (MD = − 1.84, 95%CI: − 3.42 to − 0.26, *P* = 0.002; Fig. 4), with high heterogeneity across studies (chi^2^ = 28.44, *P* < 0.00001, I^2^ = 89%; Fig. 4). The pooled MD for macular whole enface deep VD showed high heterogeneity (chi^2^ = 91.57, *P* < 0.00001, I^2^ = 97%; Fig. 4) and was lower in eyes with non-ocular BD, although the difference was not significant (MD = − 2.19, 95%CI: − 5.66 to 1.28, *P* = 0.22; Fig. 4).

### Foveal VD analysis in BD patients and controls

**Figure 5:**
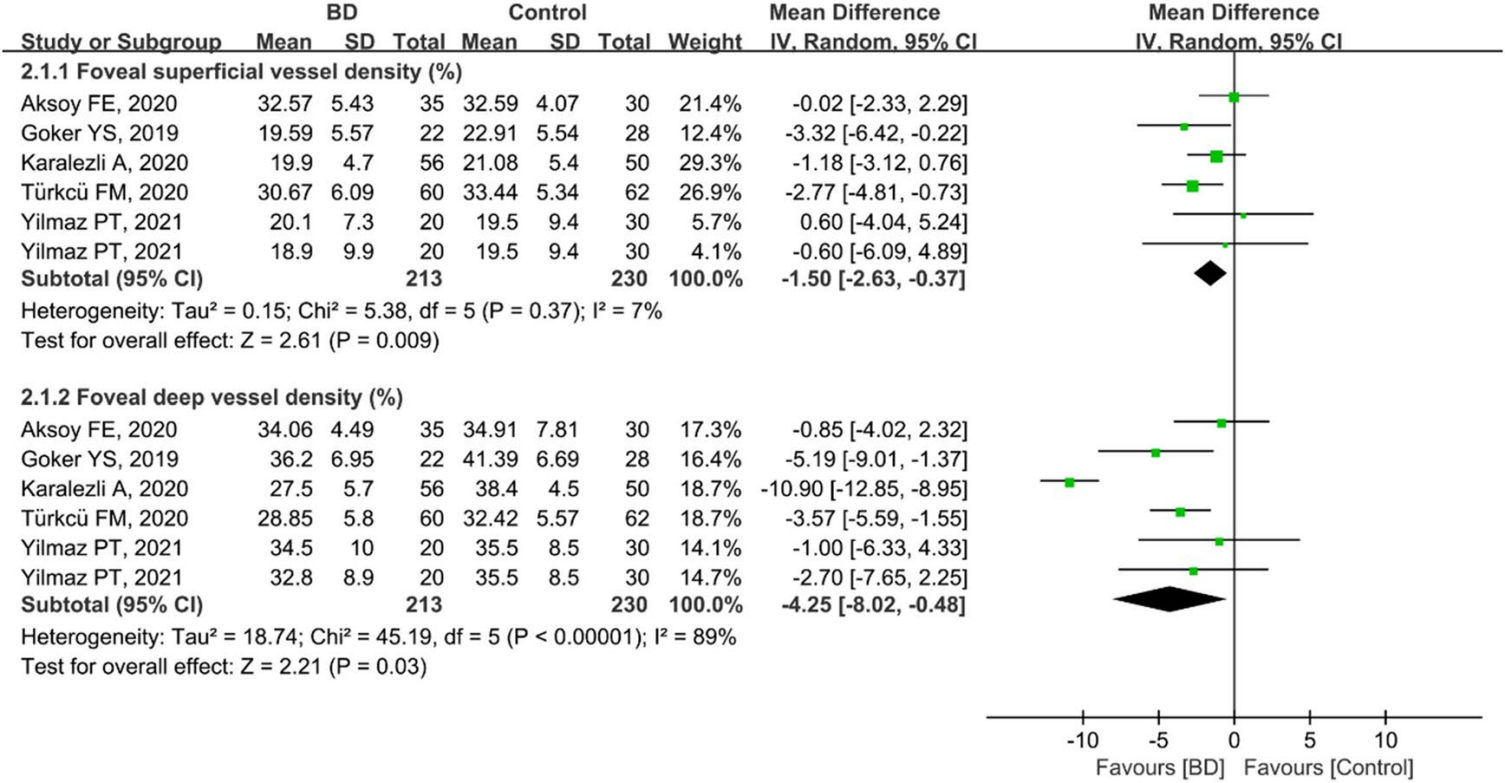
Forest plot for foveal superficial and deep vessel density between BD and control groups.

**Figure 6:**
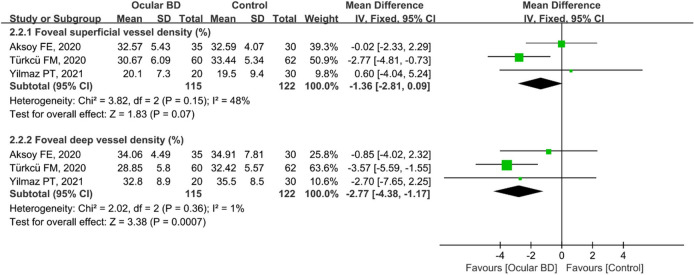
Forest plot analysis of foveal superficial and deep vessel density between eyes with ocular BD and the controls.

A total of 443 eyes (213 eyes in the BD group and 230 eyes in the control group) in five studies were included in the analysis of superficial and deep foveal VD. The pooled foveal superficial VD (MD: − 1.50, 95%CI: − 2.63 to − 0.37, *P* = 0.009; Fig. 5) and deep VD (MD: − 4.25, 95%CI: − 8.02 to − 0.48, *P* = 0.03; Fig. 5) were significantly lower in the BD group than in the control group and were associated with mild (chi^2^ = 5.38, *P* = 0.37, I^2^ = 7%; Fig. 5) and high (chi^2^ = 45.19, *P* < 0.00001, I^2^ = 89%; Fig. 5) heterogeneity across studies, respectively. Subgroup analyses in three studies showed that the pooled MD for foveal superficial VD was lower in eyes with ocular BD (MD = − 1.36, 95%CI: − 2.81 to 0.09, *P* = 0.07; Fig. 6), with mild heterogeneity across studies (chi^2^ = 3.82, *P* = 0.15 I^2^ = 48%; Fig. 6). In addition, foveal deep VD was significantly lower in eyes with non-ocular BD (MD: − 2.77, 95%CI: − 4.38 to 1.17, *P* = 0.0007; Fig. 6), and the related studies showed nearly minimal heterogeneity (chi^2^ = 2.02, *P* = 0.36, I^2^ = 1%; Fig. 6).

**Figure 7:**
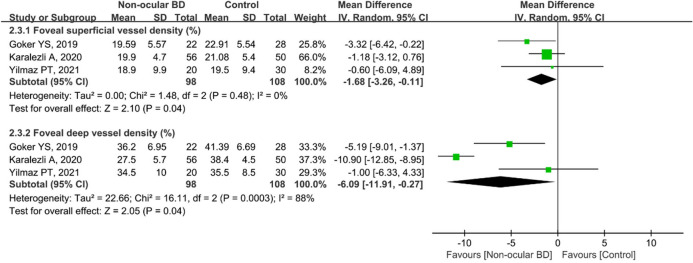
Forest plot analysis of foveal superficial and deep vessel density in non-ocular BD patients and controls.

Furthermore, three studies were analyzed for foveal VD in the non-ocular BD and control groups. The difference was significant between the two groups in terms of foveal superficial VD (MD: − 1.68, 95%CI: − 3.26 to − 0.11, *P* = 0.04; Fig. 7), and there was no heterogeneity among the studies for this parameter (chi^2^ = 1.48, *P* = 0.48, I^2^ = 0%; Fig. 7). The summary MD for foveal deep VD was significantly lower in non-ocular BD participants than in controls (MD: − 6.09, 95%CI: − 11.91 to − 0.27, *P* = 0.04; Fig. 7); however, there was high heterogeneity across the studies (chi^2^ = 16.11, *P* = 0.0003, I^2^ = 88%; Fig. 7).

### Parafoveal VD in patients with BD and controls

**Figure 8:**
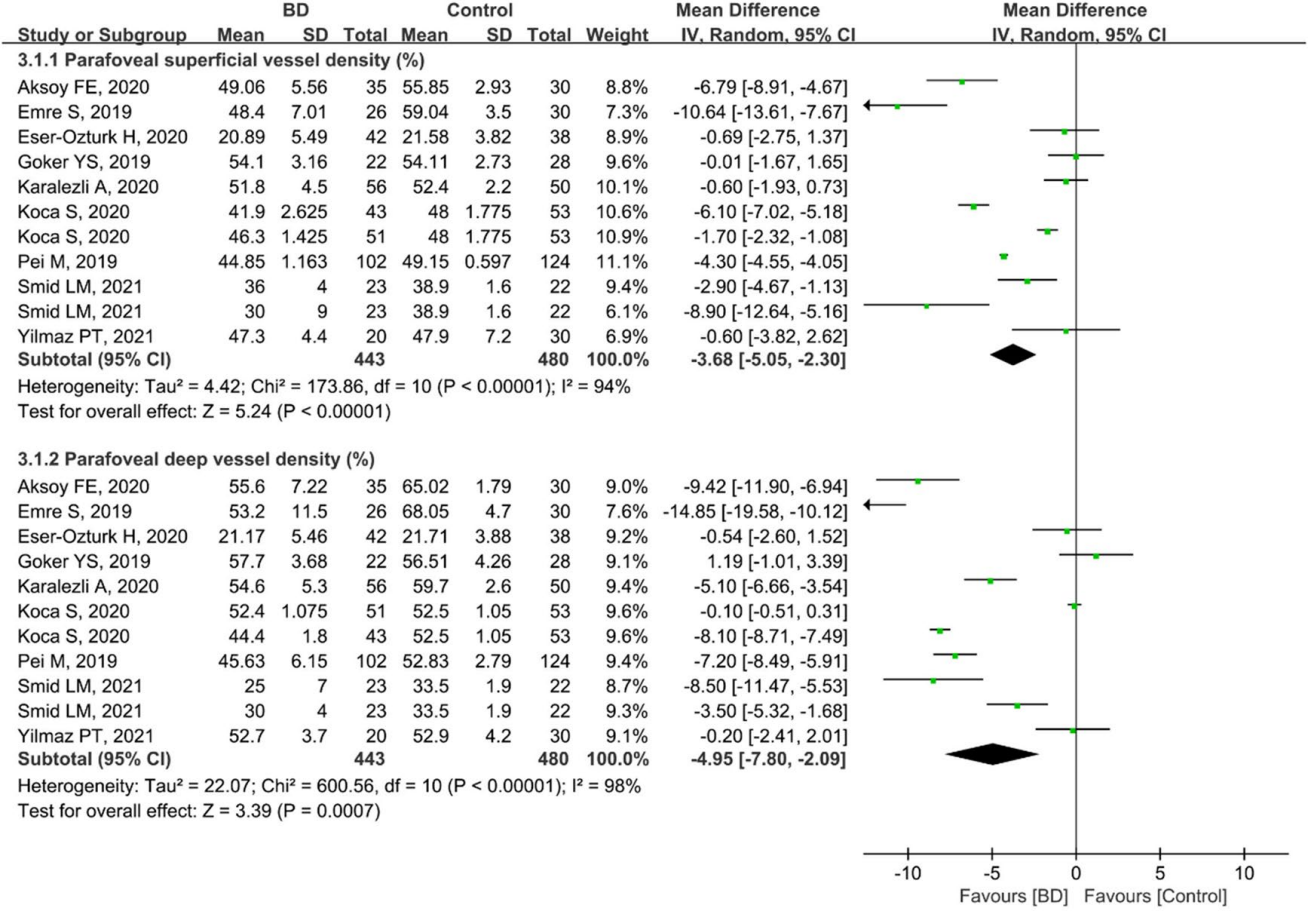
Forest plot for parafoveal superficial and deep vessel density between BD and control groups.

**Figure 9:**
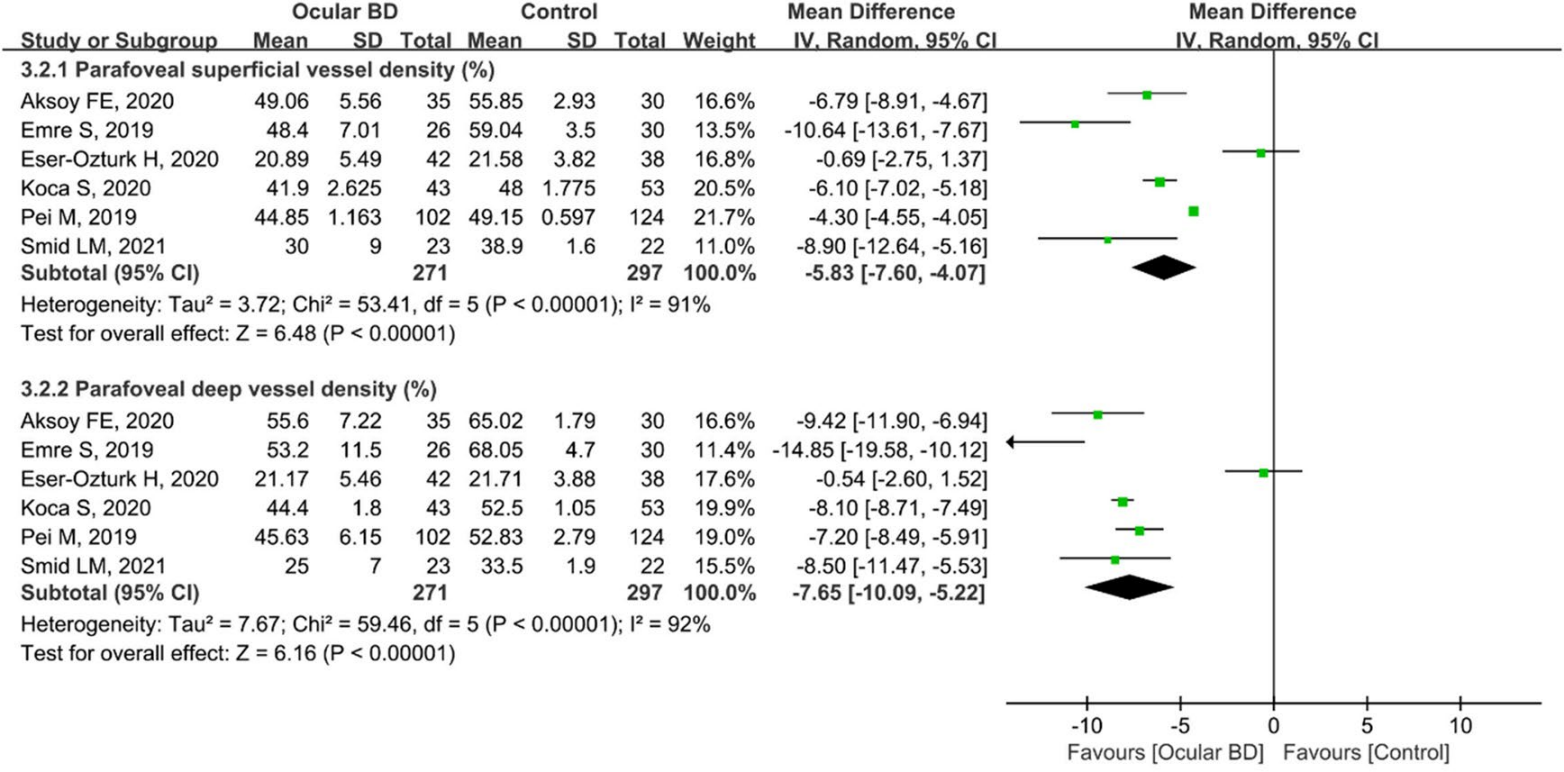
Forest plot analysis of parafoveal superficial and deep vessel density between eyes with ocular BD and the controls.

Regarding parafoveal VD, 923 eyes (443 eyes in the BD group and 480 eyes in the control group) in nine studies were included in the analysis. The pooled results for parafoveal superficial VD (MD: − 3.68, 95%CI: − 5.05 to − 2.30, *P* < 0.00001; Fig. 8) and deep VD (MD: − 4.95, 95%CI: − 7.80 to − 2.09, *P* = 0.0007; Fig. 8) were significantly lower in the BD group than in the control group, with high heterogeneity across studies (superficial VD: chi^2^ = 173.86, *P* < 0.00001, I^2^ = 94; deep VD: chi^2^ = 600.56, *P* < 0.00001, I^2^ = 98%; Fig. 8). Subgroup analyses in six studies revealed that pooled results for parafoveal superficial VD (MD: − 5.83, 95%CI: − 7.60 to − 4.07, *P* < 0.00001) and deep VD (MD: − 7.65, 95%CI: − 10.09 to − 5.22, *P* < 0.00001) were significantly lower in ocular BD patients than in controls (Fig. 9), and these studies had high heterogeneity (superficial VD: chi^2^ = 53.41, *P* < 0.00001, I^2^ = 91%; deep VD: chi^2^ = 59.46, *P* < 0.00001, I^2^ = 92%) (Fig. 9).

**Figure 10:**
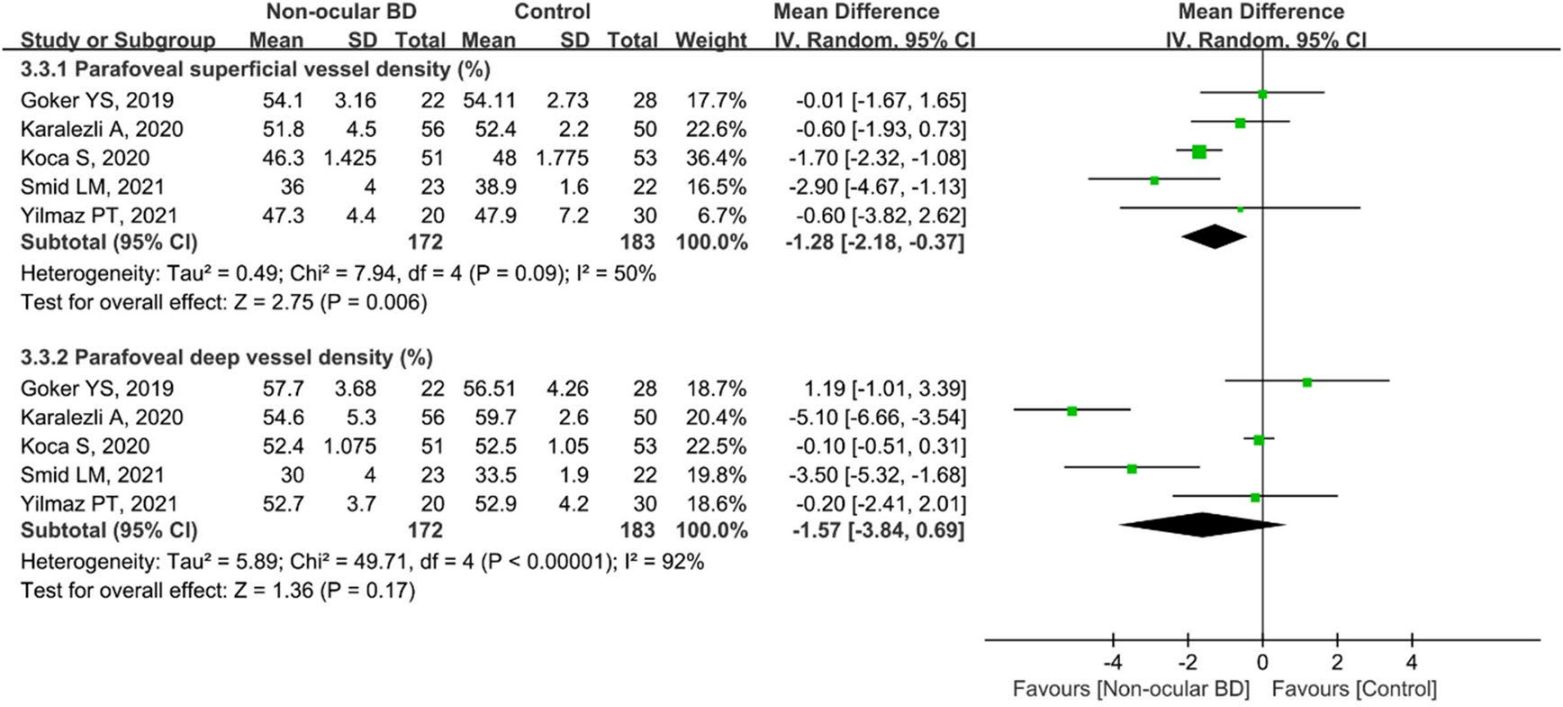
Forest plot analysis of parafoveal superficial and deep vessel density in non-ocular BD patients and controls.

In addition, five studies including 355 eyes (172 eyes in the non-ocular BD group and 183 eyes in the control group) compared the parafoveal superficial VD between the groups. The difference was significant between the two groups (MD = − 1.28, 95% CI: − 2.18 to − 0.37, *P* = 0.006; Fig. 10), and there was moderate heterogeneity across the studies (chi^2^ = 7.94, *P* = 0.09, I^2^ = 50%; Fig. 10). The pooled result for parafoveal deep VD revealed substantial heterogeneity (chi^2^ = 49.71, *P* < 0.00001, I^2^ = 92%; Fig. 10), and parafoveal deep VD was lower in eyes with non-ocular BD, although the difference was not significant (MD: − 1.57, 95%CI: − 3.84 to 0.69, *P* = 0.17; Fig. 10).

### FAZ analysis in patients with BD and controls

**Figure 11:**
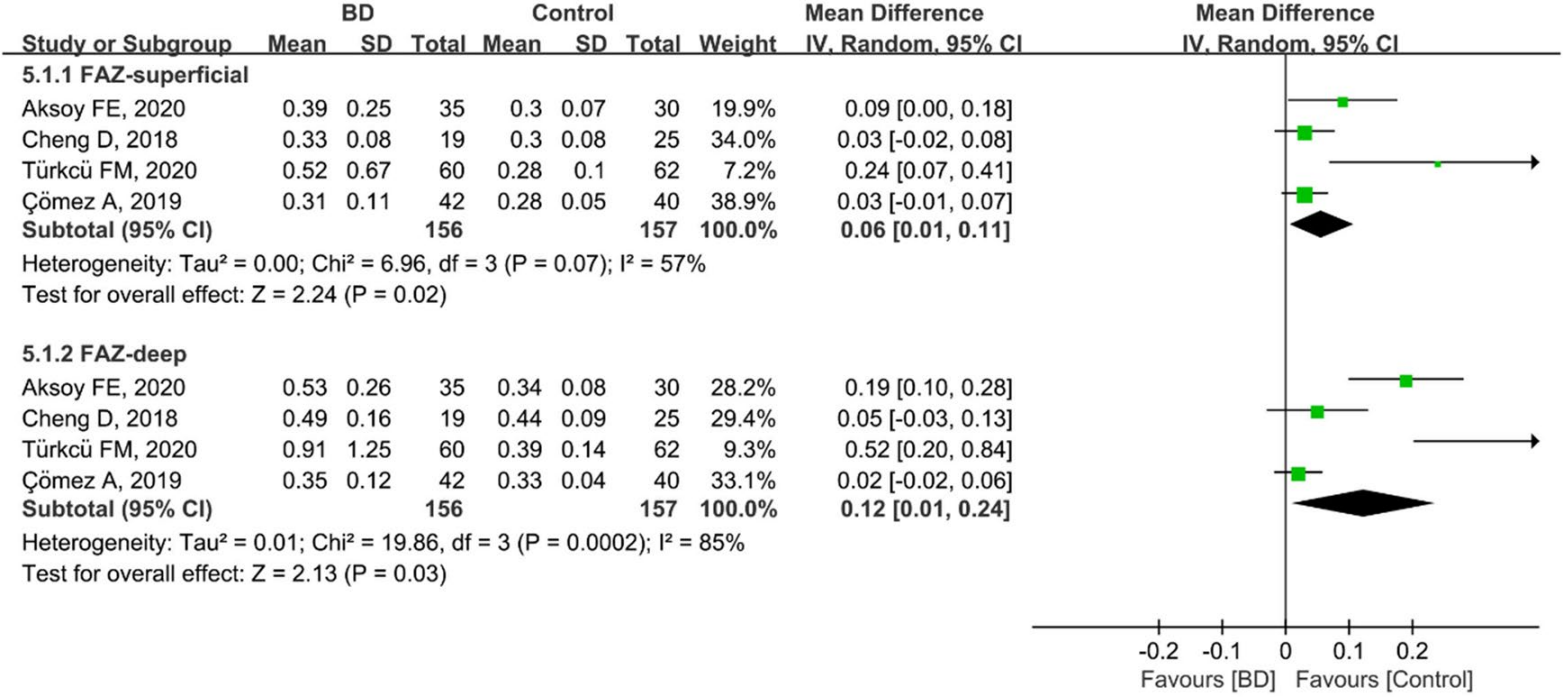
Forest plot for FAZ superficial and deep in eyes with BD and controls.

Four studies including 313 eyes (156 eyes in the BD group and 157 eyes in the control group) compared superficial and deep FAZ between these two groups. Among these studies, eyes with BD had significantly larger superficial (MD: 0.06, 95%CI: 0.01 to 0.11, *P* = 0.02; Fig. 11) and deep (MD: 0.12, 95%CI: 0.01 to 0.24, *P* = 0.03; Fig. 11) FAZs, with moderate and high heterogeneity (superficial FAZs: chi^2^ = 6.96, *P* = 0.07, I^2^ = 57%; deep FAZs: chi^2^ = 19.86, *P* = 0.0002, I^2^ = 85%; Fig. 11).

### Publication bias

Funnel plots summarized the potential publication bias of macular whole enface superficial and deep VD, foveal superficial and deep VD, parafoveal superficial and deep VD and superficial and deep FAZ among the included literatures. The results showed that the distribution of articles was not an obvious asymmetry, revealing no remarkable publication bias (Supplementary Figure S1–S4).

## Discussion

BD is a chronic systemic inflammatory vasculitis with unknown etiology characterized by recurrent oral ulcers, genital ulcers, skin lesions, and ocular lesions^36,37^, and ocular involvement is characterized by posterior uveitis or uveitis. OCTA has been used to investigate retinal microvascular changes in uveitis^15^. Although available studies have revealed that retinal VDs change in eyes with BD, the related results are inconsistent. For instance, some studies found significantly lower foveal superficial and deep VDs in patients with BD^16,17^, while others demonstrated no difference in foveal superficial vascular density^19,20^. Given these conflicting results, we conducted this meta-analysis to systemically analyze retinal vessel features in patients with BD. To our knowledge, this meta-analysis is the first to explore retinal microvascular features using OCTA in patients with BD and control individuals. Thirteen available studies, including 599 eyes with BD and 622 control eyes, were analyzed. The present meta-analysis revealed that macular whole enface VD, foveal VD, and parafoveal VD were significantly lower in eyes with BD, and the FAZ was significantly larger in patients with BD.

To date, the pathogenesis of ocular vascular changes in BD patients remains unclear. The pathogenesis of vascular alterations can be explained by endothelial dysfunction, vascular inflammation, coagulation abnormalities, and changes in retrobulbar hemodynamics^38–40^. Some studies have observed that endothelial function is impaired^41,42^ and plasma angiopoietin-1 (Ang-1) levels are significantly lower in BD patients, especially in those with vascular involvement^43^. Ang-1 contributes to endothelial survival and shows vasculoprotective effects, and its insufficiency might elicit loss of endothelial integrity, increased permeability, and formation of perivascular inflammatory infiltration^36^. Impaired vascular endothelial cells might further facilitate immune-mediated vasculitis, leading to vascular obliteration or non-perfusion and subsequently reduced VD. In addition, other studies have shown that leukocytes, including neutrophils and mononuclear cells, infiltrate the perivascular region, mediate vascular inflammation, and induce vascular occlusion^10,44^. Notably, endothelial dysfunction and vascular inflammation contribute to thrombus formation in BD^45^. Previous studies found that there was a hypercoagulable state in patients with BD^38,42^. and the risk of developing venous thrombosis was 14-fold higher in the BD group than in the control group^46^. Indeed, the presence of prothrombotic factors, such as protein C and protein S deficiency, or factor V Leiden and prothrombin G20210A gene mutations are involved in the development of thrombosis in patients with BD^38^. Moreover, studies have demonstrated significant reductions in the central retinal artery and posterior ciliary artery flow velocities in patients with BD compared with healthy participants^47,48^. BD can affect small- and medium-sized arteries and veins simultaneously^45^. Based on the above findings, we speculated that lower VD is involved in retinal microvasculature in eyes with BD.

Uveitis, accounting for 40%–70% of cases of BD^6,7^, usually occurs within 5 years of the onset of BD^8^, causing visual impairment and blindness. Therefore, early identification of ocular vascular changes is essential for management of BD patients. In this study, we conducted a subgroup analysis of retinal VD in eyes with BD with or without ocular involvement. The pooled results showed that macular whole enface and parafoveal superficial VD and deep VD were significantly lower in eyes with BD with ocular involvement, with high heterogeneity among the studies (Figs. 3 and 9). We postulated that the quality of the included studies was relatively low, and the number of eyes was comparatively small, potentially accounting for the high heterogeneity in these results. In addition, the pooled MD in the foveal deep VD was significantly lower in eyes with BD with ocular involvement, with high homogeneity among these studies (Fig. 6). The pooled MD in foveal superficial VD was lower in patients with ocular BD than in controls (MD = − 1.36, *P* = 0.07), although the difference was not significant. We speculated that the relatively small sample size of eyes with ocular BD and controls potentially limited the power for evaluating this metric. In addition, deep foveal VD tended to be more severely affected by retinal capillary hypoperfusion or nonperfusion than superficial foveal VD^12,15^. Moreover, compared with superficial retinal capillaries, deep retinal capillaries may be more susceptible to ischemia because they are not directly connected to arterioles^33,49^. Even though substantial heterogeneity existed among the above results, our data confirmed the findings of previous studies^18,20,30,33^. Furthermore, subgroup analysis showed a significantly lower VD in the macular whole enface superficial layer, foveal superficial and deep layers, and parafoveal superficial layer in eyes with BD without ocular involvement (Figs. 4, 7, and 10). In terms of macular whole enface deep VD and parafoveal deep VD, a slightly non-significant reduction was found in eyes with non-ocular BD compared with healthy controls (Figs. 4 and 10). This noteworthy phenomenon may be due to projection artifacts, which are caused by superficial vessels projecting shadows onto deeper layers of the retina^50^. Another important explanation may be the relatively small number of eyes in the two groups. Moreover, differences in disease duration and patient characteristics may contribute to this discrepancy^34^. Our results are consistent with those of several studies that revealed a significant reduction in VD in the macular whole enface superficial and deep layers, foveal superficial and deep layers, and parafoveal superficial and deep layers in eyes with non-ocular BD^16,17,32^.

The lower foveal VD may consequently lead to a larger FAZ. However, inconsistent results on the FAZ between eyes with BD and controls have been reported in previous studies^17,30,32,33^. In our study, we demonstrated significantly larger FAZs in superficial and deep layers in eyes with BD than in controls, with moderate and high heterogeneity across the studies, respectively (Fig. 11). The source of the high heterogeneity arises primarily from the relatively small sample size. Manual delineation of the FAZ by researchers as well as different segmentation methods for FAZ measurement may also explain this bias^51,52^. In addition, relative variability of the FAZ area in healthy individuals has been observed in previous studies^53,54^. Furthermore, the authors hypothesized that an enlarged FAZ is attributed to repeated ocular attacks aggravating retinal ischemia^30,33^. Although heterogeneity existed for this parameter, our pooled results confirmed the findings in previous studies that compared the FAZ in eyes between BD patients and controls^18,30^. Further prospective and larger cohort studies are needed to verify our results.

Our study has several limitations. First and most importantly, the number of eyes in the included articles was relatively small, and the quality of the evidence was comparatively low. Second, the pooled results should be interpreted with caution because statistical heterogeneity appeared across the individual studies. Third, the source of heterogeneity could not be fully elucidated because of insufficient data to perform a meta-regression. Fourth, this study was not registered in the PROSPERO database. However, no corresponding systematic review registration was found in the database. To further verify our findings, prospective longitudinal studies with larger sample sizea should be conducted to assess retinal microvasculature alterations in patients with BD in the future.

In conclusion, our meta-analysis found that macular whole enface VD, foveal VD, and parafoveal VD were lower in eyes with BD, and FAZ was larger in patients with BD. Our findings suggest that OCTA can help clinicians to diagnose and monitor the status of patients with BD early.

## Supplementary Information

Supplementary Information.

## Data Availability

All relevant data supporting the conclusions of this study are included in the article.
