# Supplementary material for: Retinal microvasculature features in patients with Behcet’s disease: a systematic review and meta-analysis
Source: Sci Rep. 2022 Jan 14;12:752. doi: 10.1038/s41598-021-04730-6 (PMC8760269; doi:10.1038/s41598-021-04730-6)

**Supplementary Table S1.** Detailed electronic search strategy for systematic review.

| Electronic databases searched | PubMed (http://www.ncbi.nlm.nih.gov/PubMed/),  Embase (http://www.embase.com),  Cochrane Library (http://www.thecochranelibrary.com/), and Web of Science (http://webofknowledge.com/WOS) |
| --- | --- |
| Search terms for Pubmed was conducted to identify qualified literature from inception to April 8, 2021(similar strategies were applied to other electronic databases) | ((((OCTA) OR (OCT angiography)) OR (optical coherence tomography angiography)) OR (optical coherence tomographic angiography)) AND ((((Behcet’s disease) OR (Behcet disease)) OR (Behcet’s syndrome)) OR (BD)) |
| Limitation | Articles written in English were considered eligible. |
| Other sources | The references of included articles were searched and reviewed to recognize any relevant literatures. |

**Supplementary Figure S1.** A funnel plot of macular whole enface superficial and

deep VD showing no significant publication bias. SE = standard error, MD = mean difference.


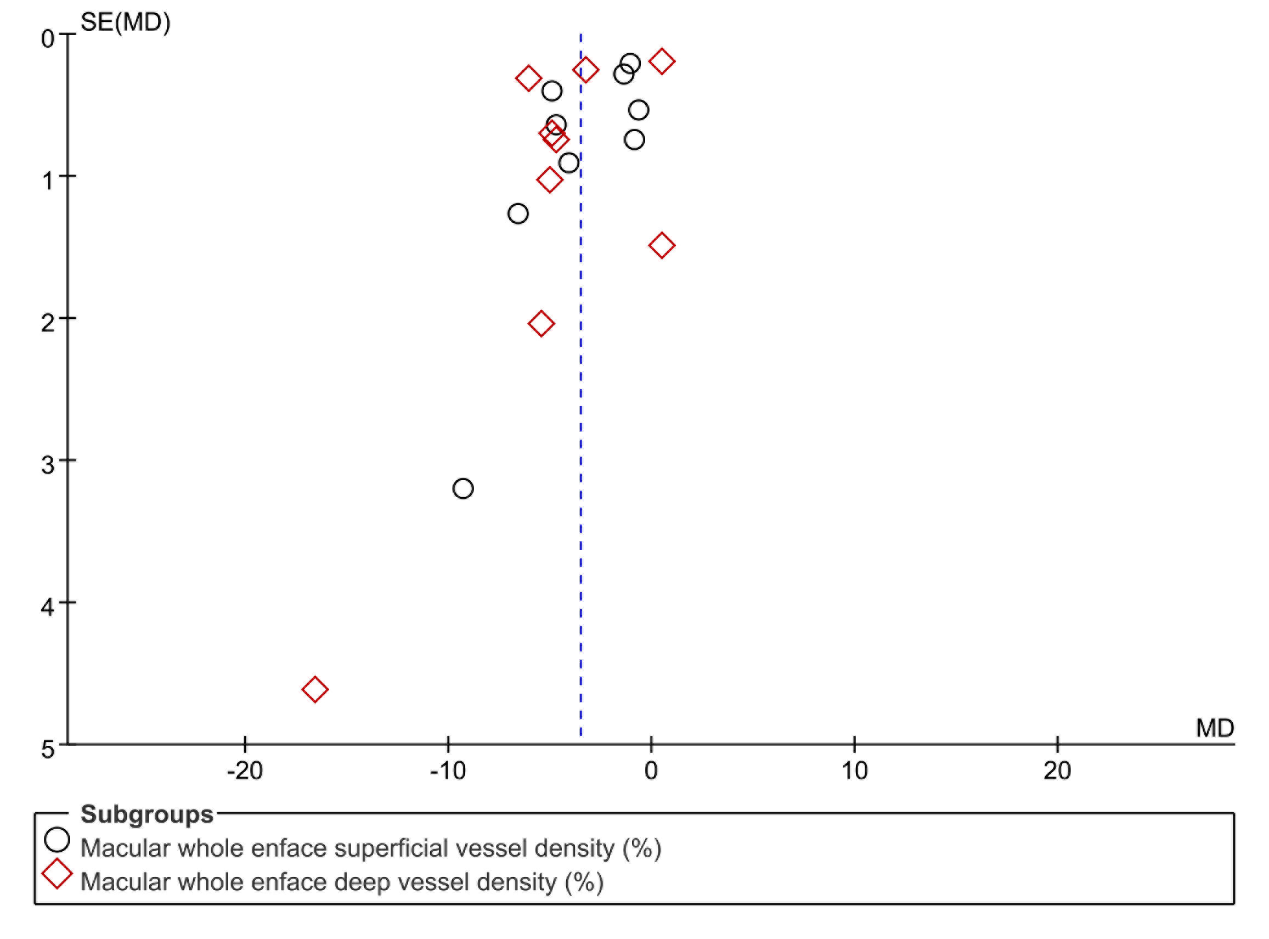


**Supplementary Figure S2.** A funnel plot of foveal superficial and deep VD revealing no substantial publication bias. SE = standard error, MD = mean difference.


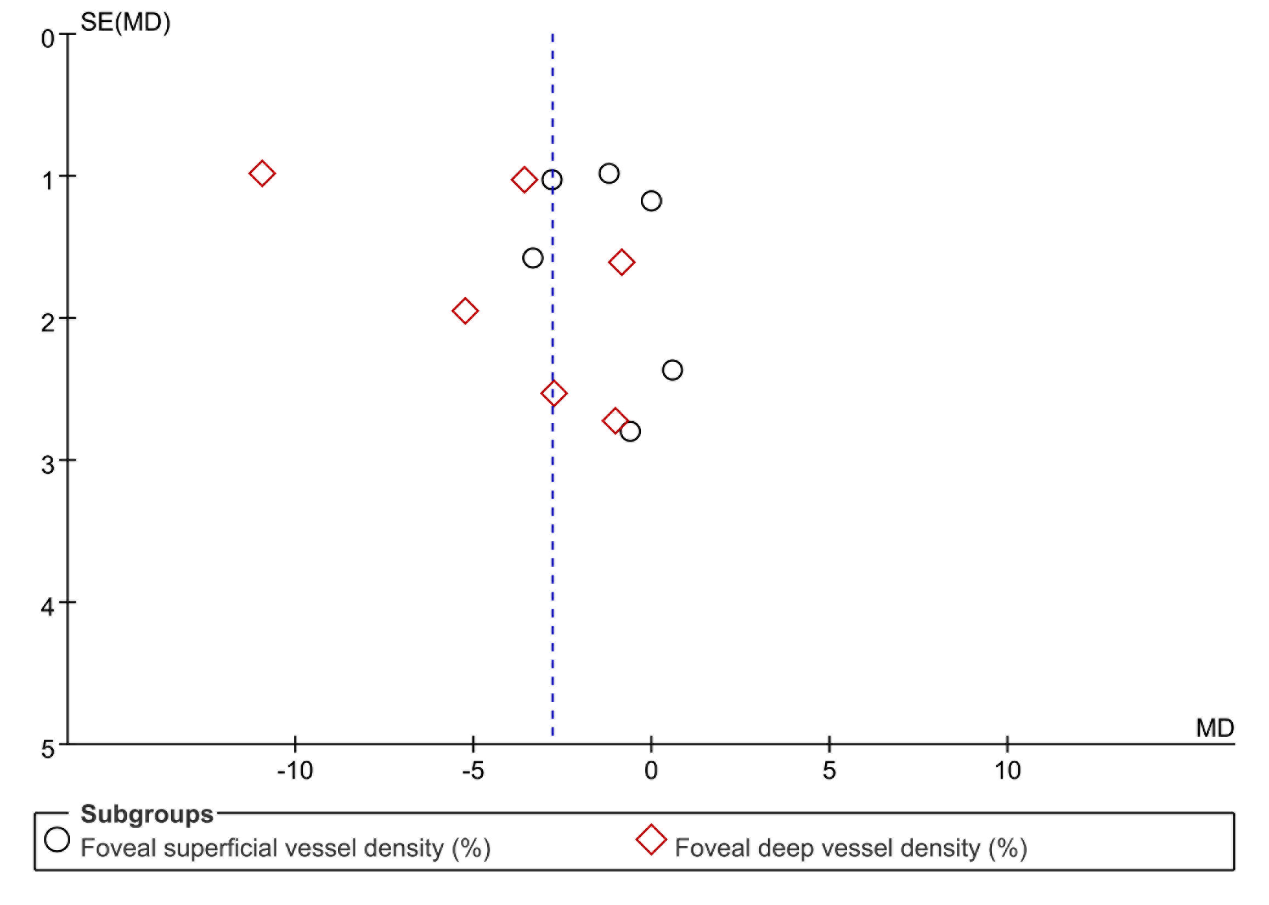


**Supplementary Figure S3.** A funnel plot of parafoveal superficial and deep VD indicating no remarkable publication bias. SE = standard error, MD = mean difference.


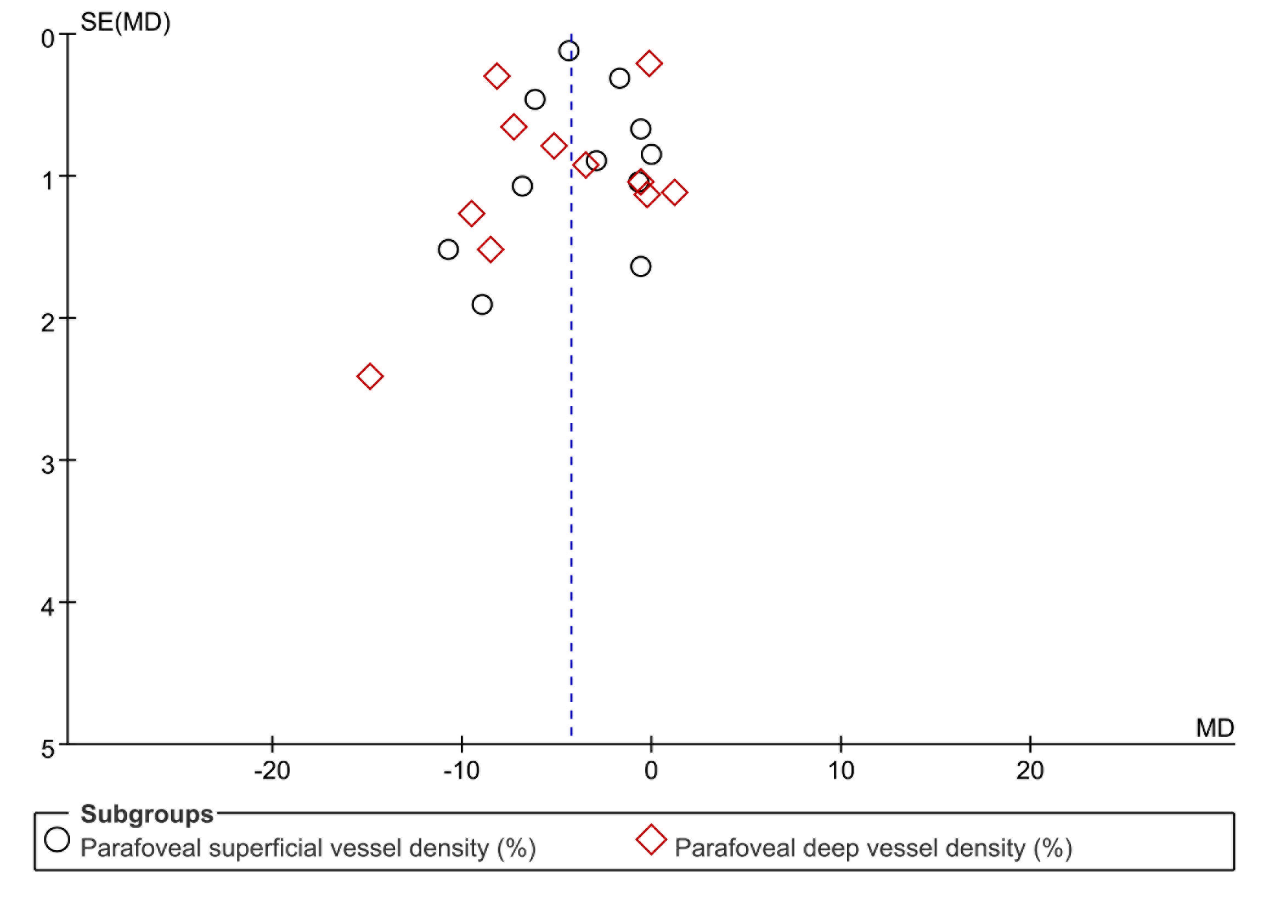


**Supplementary Figure S4.** A funnel plot of superficial and deep FAZ demonstrating no significant publication bias. SE = standard error, MD = mean difference.


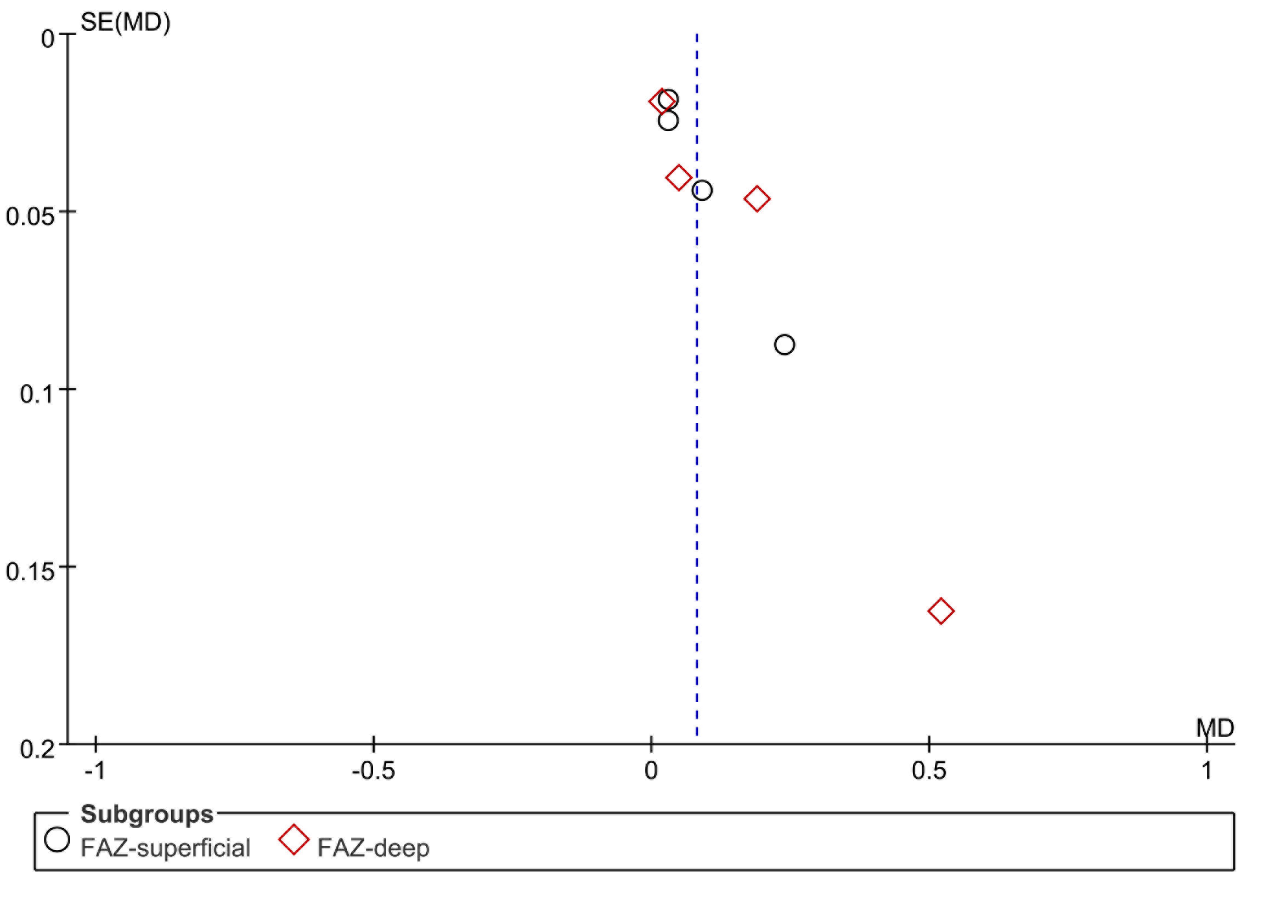

Supplement: Supplementary file 1 — Supplementary Information. [file 41598_2021_4730_MOESM1_ESM.docx]
